# Supplementary material for: Potent pollen gene regulation by DNA glycosylases in maize
Source: Nat Commun. 2024 Sep 27;15:8352. doi: 10.1038/s41467-024-52620-y (PMC11436724; doi:10.1038/s41467-024-52620-y)
Supplement: Supplementary file 1 — Supplementary Information [file 41467_2024_52620_MOESM1_ESM.pdf]

Potent pollen gene regulation by DNA glycosylases in maize

Supplementary Figures 1-9 and Supplementary Table 1

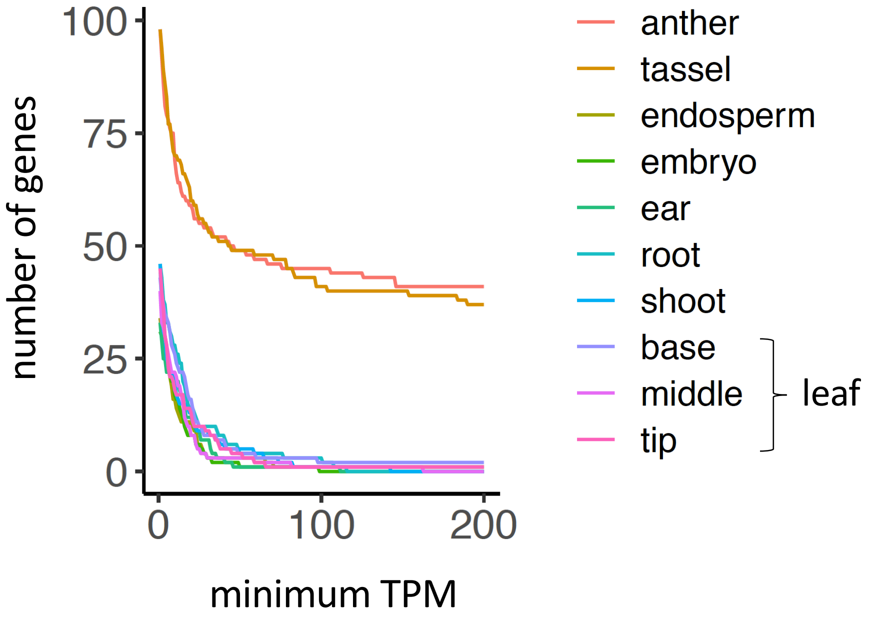

Supplemental Fig. 1: High expression of genes with TE-like methylation in pollen-containing tissues.
The Y axis indicates the number of genes with TE-like methylation, plotted relative to the minimum expression thresholds (X axis) in each tissue type. This analysis only includes genes that are present at syntenic positions in B73 and the other 25 NAM founder genomes (core genes) and whose coding DNA sequence (CDS) does not overlap at all with TE annotations.

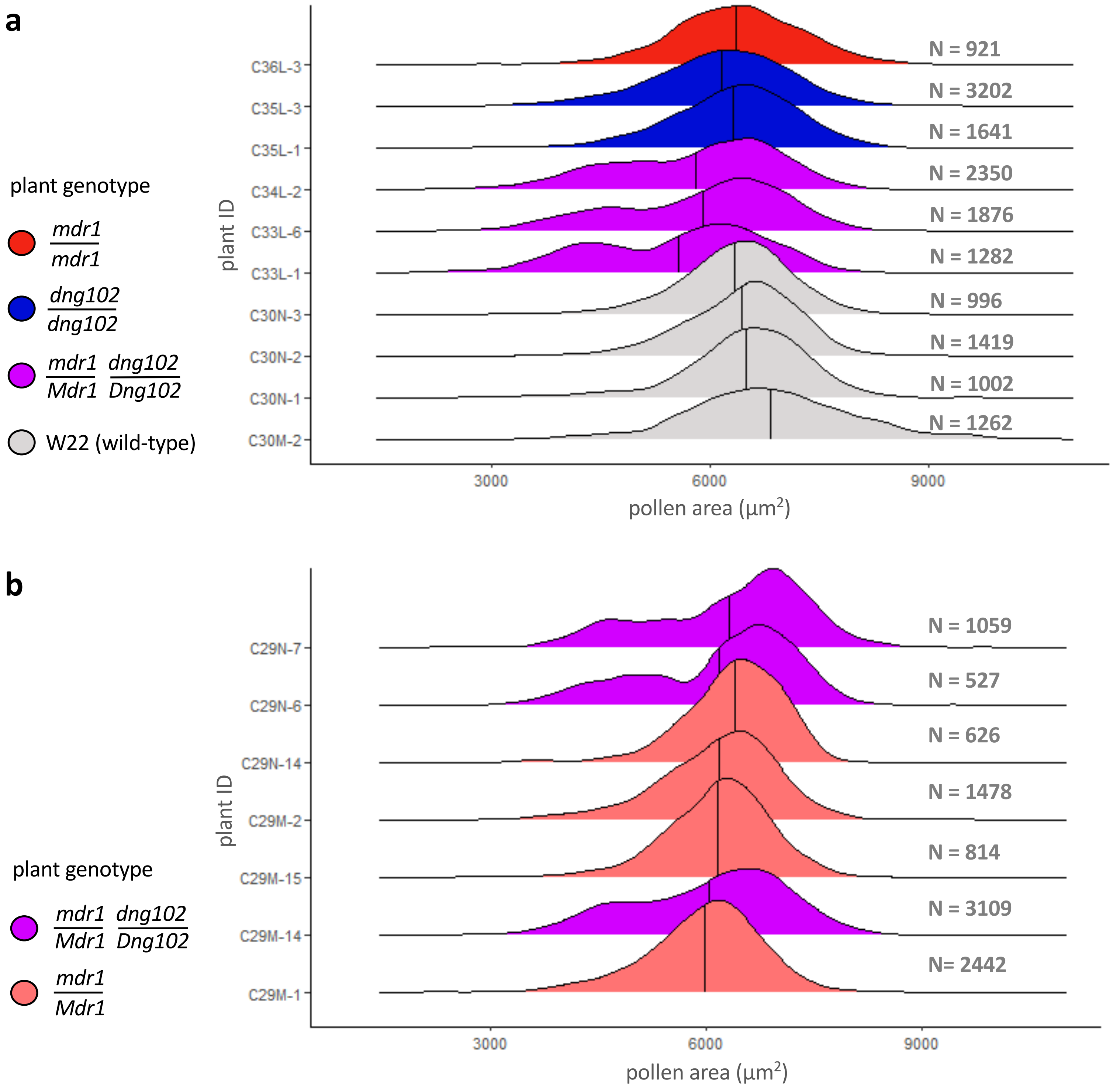

Supplementary Fig. 2: Small pollen phenotype in pollen segregating *mdr1* and *dng102* mutations.
a Distribution of 2-D pollen grain areas based on microscope image analysis. Each distribution is derived from pollen grains from a single plant whose genotype is indicated by the color on the left. All plants are near isogenic with W22. Plant IDs are in the format "family"-"sibling number". Capital allele indicates WT, lowercase mutant. The *mdr1* allele is the original Kermicle allele, and *dng102* allele is the Q235 allele. The number of pollen grains analyzed for each plant is indicated to the right.
b Distribution of 2-D pollen grain areas based on microscope image analysis from plants that are near isogenic with W22, as in **a**. All plants are siblings. The *mdr1* allele is EMS4-06835d, and *dng102* is the Q235 allele.

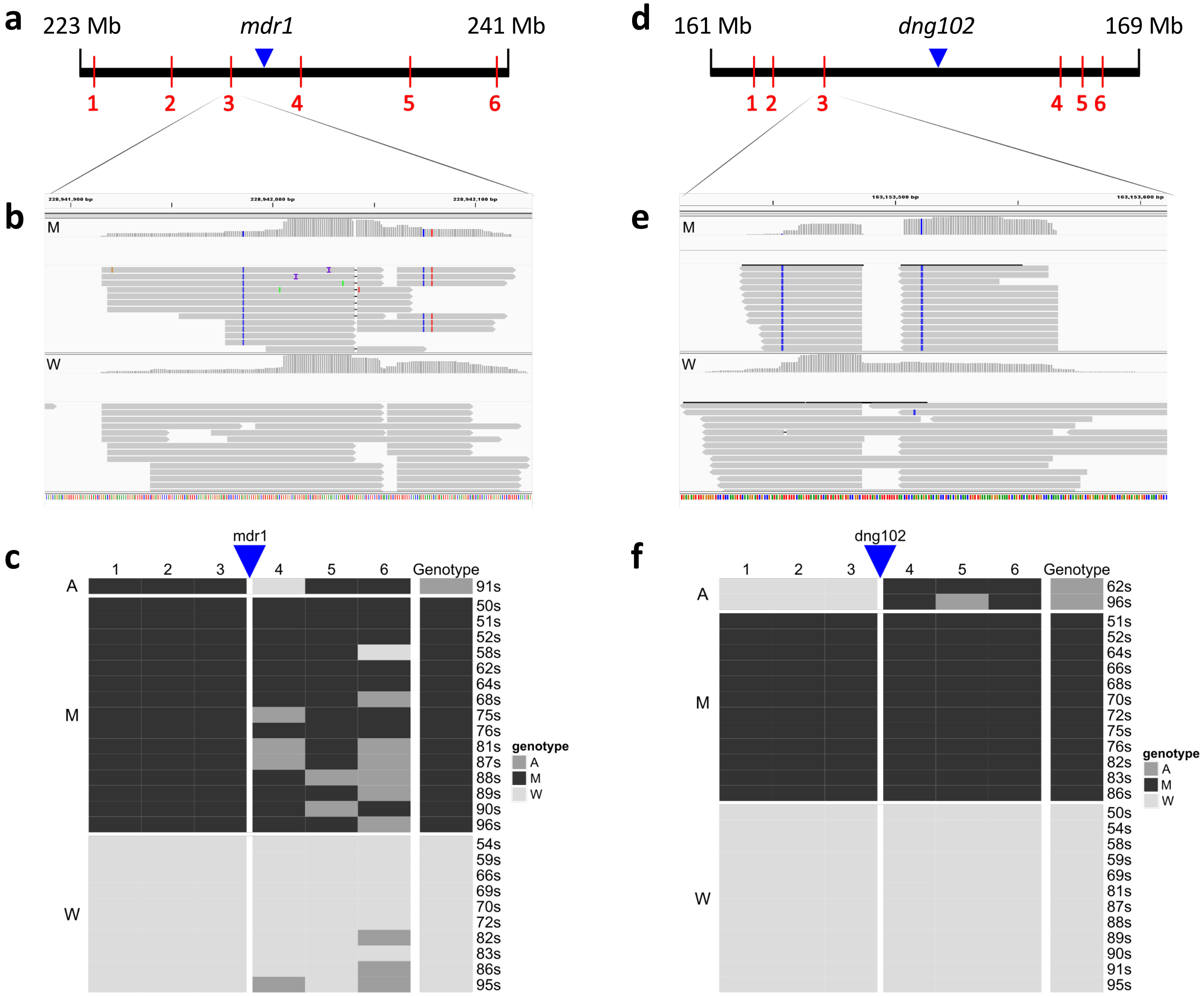

Supplementary Fig. 3: Pollen genotyping.
a, d Map of DNG and sentinel gene locations on chromosome 4 (*mdr1*) (**a**) and chromosome 5 (*dng102*) (**d**). *mdr1*-linked sentinel genes are (1) Zm00004b023798, (2) Zm00004b023832, (3) Zm00004b023880, (4) Zm00004b023919, (5) Zm00004b024000, and (6) Zm00004b024077. *dng102*-linked sentinel genes are (1) Zm00004b013764, (2) Zm00004b013769, (3) Zm00004b013785, (4) Zm00004b013866, (5) Zm00004b013875, and (6) Zm00004b013879. Sentinel genes were inherited from B73 along with *mdr1* and *dng102* and have sufficient expression in pollen to detect SNPs relative to the W22 reference genome. WT *Mdr1* and *Dng102* alleles and the rest of the genome are from W22.
b, e IGV browser images showing B73 SNPs (marked blue and red) in sequencing reads (gray arrows) at example sentinel genes in mutant (M) but not in WT (W), linked to *mdr1* (**b**) or *dng102* (**e**).
c, f Summary of pollen grain genotypes at each DNG locus, (**c**) for *mdr1*, (**f**) for *dng102*, based on SNP calling (left, M = mutant; W = wild-type). Each row represents a single pollen transcriptome, each column a sentinel gene. Genotypes at sentinel genes: black is mutant (M), light grey is wild-type (W). In cases where no reads were detected at the SNP sites at a sentinel gene, it was disregarded for that transcriptome (dark gray, A = ambiguous). In three pollen grains, the sentinel genes nearest to *dng102* or *mdr1* had conflicting genotypes, possibly due to recombination between the sentinel genes; these pollen grains were scored as ambiguous genotypes at the respective loci.

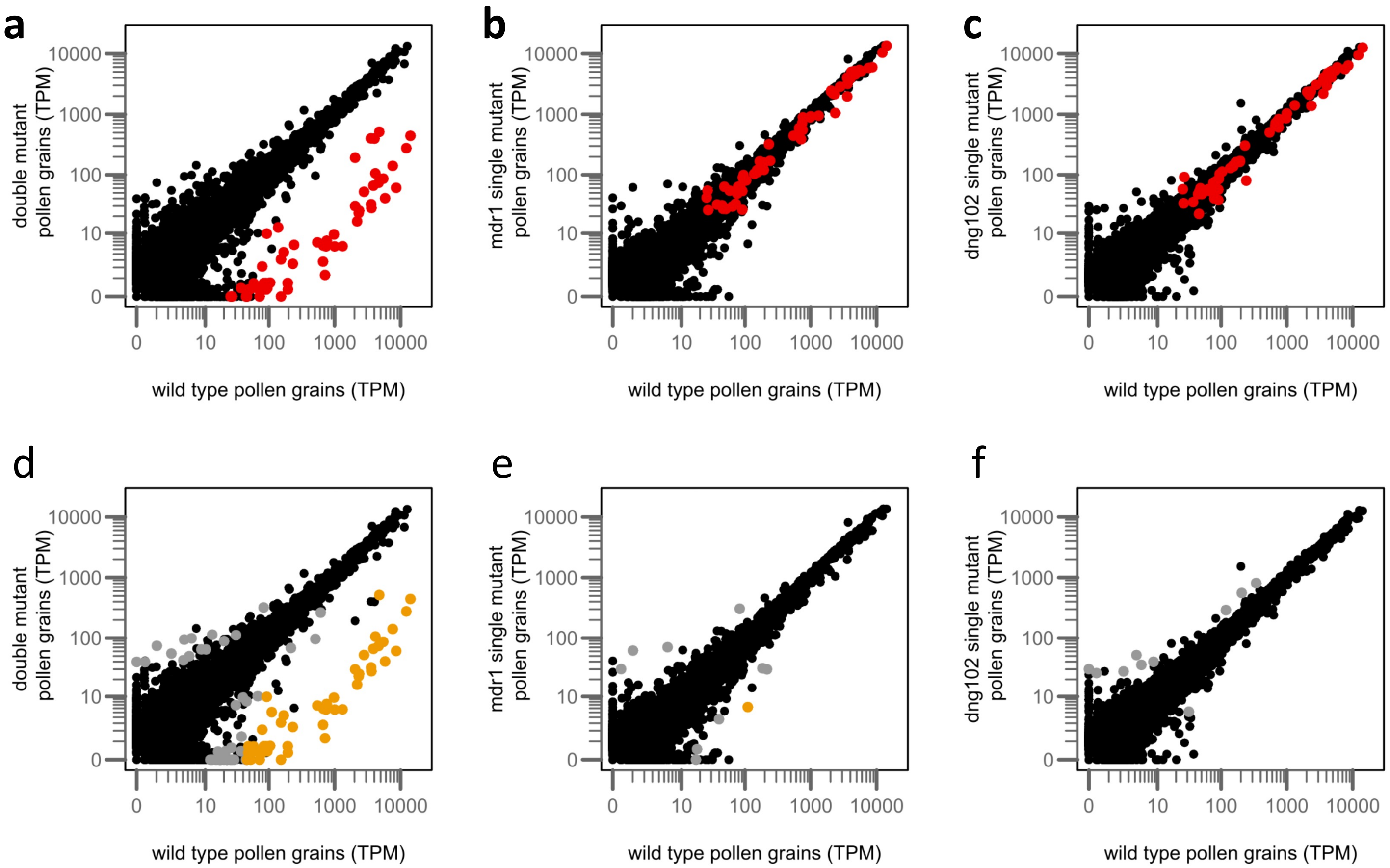

Supplementary Fig. 4: Expression analysis of *mdr1* and *dng102* single mutant pollen.

a-c. Expression of DEGs (highlighted in red) from Figure 2A, plotted in WT vs. double mutant (**a**); WT vs. *mdr1* single mutant (**b**); and WT vs. *dng102* single mutant (**c**).

d-f. Differential expression of individual genes in WT vs. double mutant (**d**), WT vs. *mdr1* single mutant (**e**), and WT vs. *dng102* single mutant (**f**). Strongly differentially expressed genes (adjusted p-value ≤ 0.05; ≥ 8-fold change in expression; ≥ 10 average UMI counts, as estimated by DESeq2) are highlighted in orange. Weakly differentially expressed genes (adjusted p-value ≤ 0.05; ≥ 2-fold change in expression) are highlighted in gray.

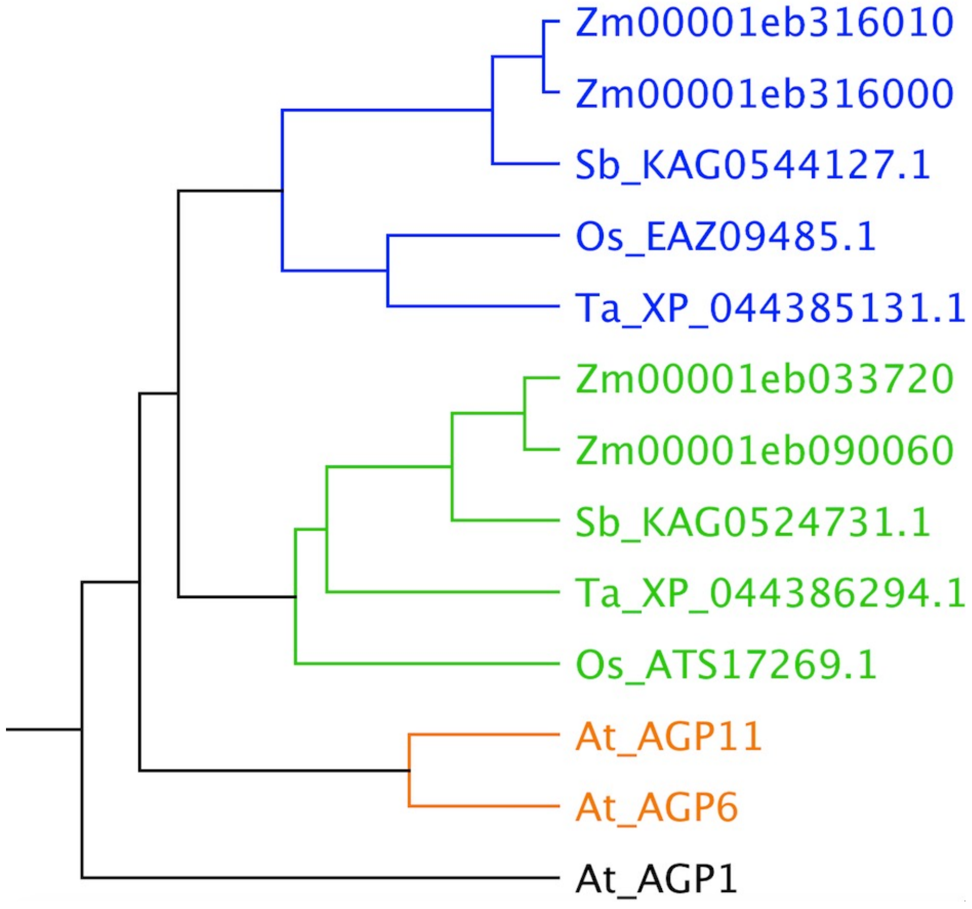

Supplementary Fig. 5: Protein tree of arabinogalactan protein (AGP) homologs. The tree was generated using UPGMA based on sequence similarity. Zm indicates *Zea mays*, Sb *Sorghum bicolor*, Os *Oryza sativa*, Ta *Triticum aestivum,* and At *Arabidopsis thaliana*.

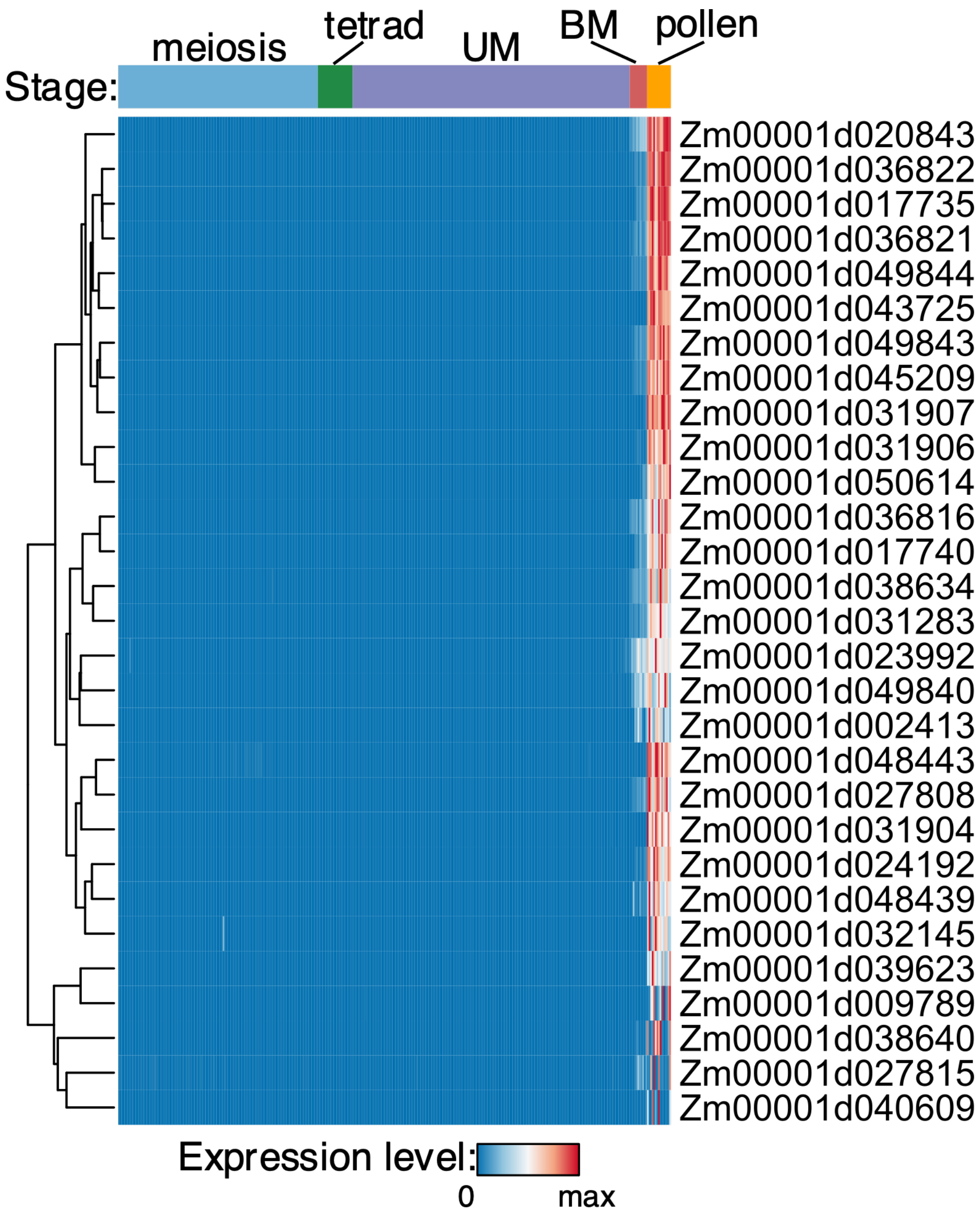

Supplementary Fig. 6: Expression timecourse of differentially expressed genes (DEGs)
UM = unicellular microspore, BM = bicellular microspore. Data are from B73 single-cell transcriptomes (Nelms and Walbot 2022). Each column represents a single pollen grain or pollen precursor. Gene names are listed as B73 v4 names because the published expression analysis was carried with v4 annotations. Mappings used between v4 and v5 are shown in Supplemental Table 3.

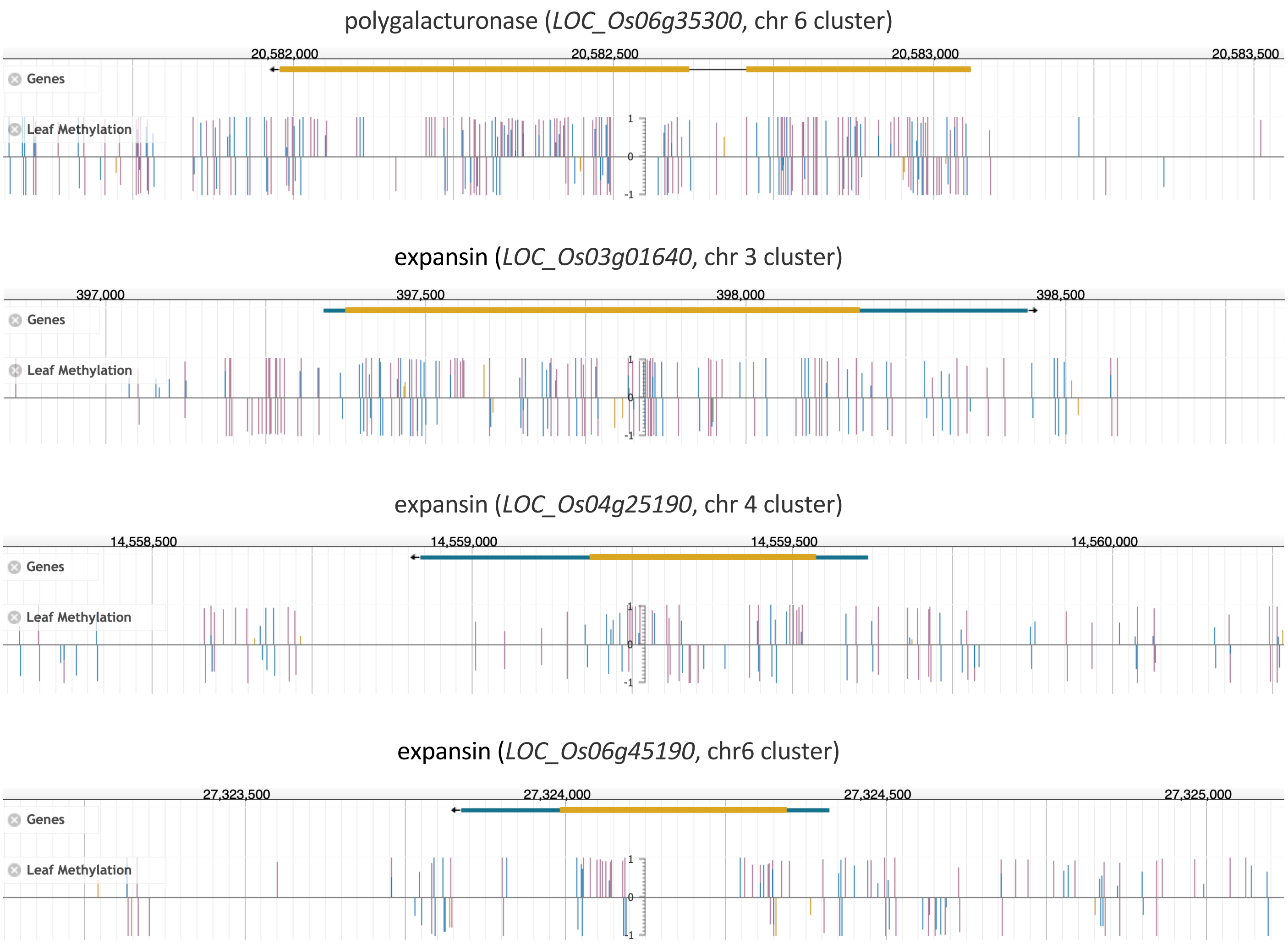

Supplementary Fig. 7: Rice pollen expansin and polygalacturonase genes with TE-like methylation.

Plant Epigenome Browser showing DNA methylation over 2 Kb regions including previously identified clustered pollen genes. CG methylation is indicated in magenta, CHG in blue, and CHH in orange. Reference coordinates are based on the Os-Nipponbare-Reference-IRGSP-1.0 assembly, v7 annotations.

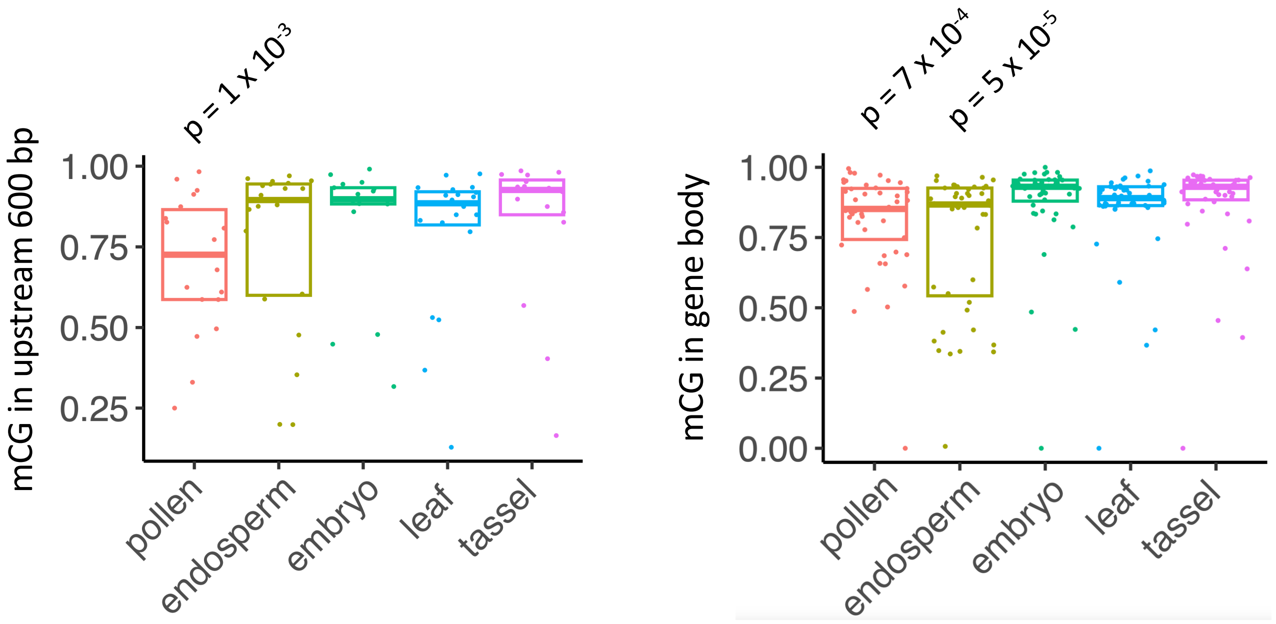

Supplementary Fig. 8: Demethylation in pollen and endosperm.
The Y-value for each dot represents the average mCG value for the 600 bp upstream of a gene (left) or gene body (right). Only the 44 of 56 methylated pollen genes (MPGs) present in both the W22 and B73 genome annotations are included. Boxes indicate interquartile ranges. P-values from one-tailed Wilcoxon signed rank tests.

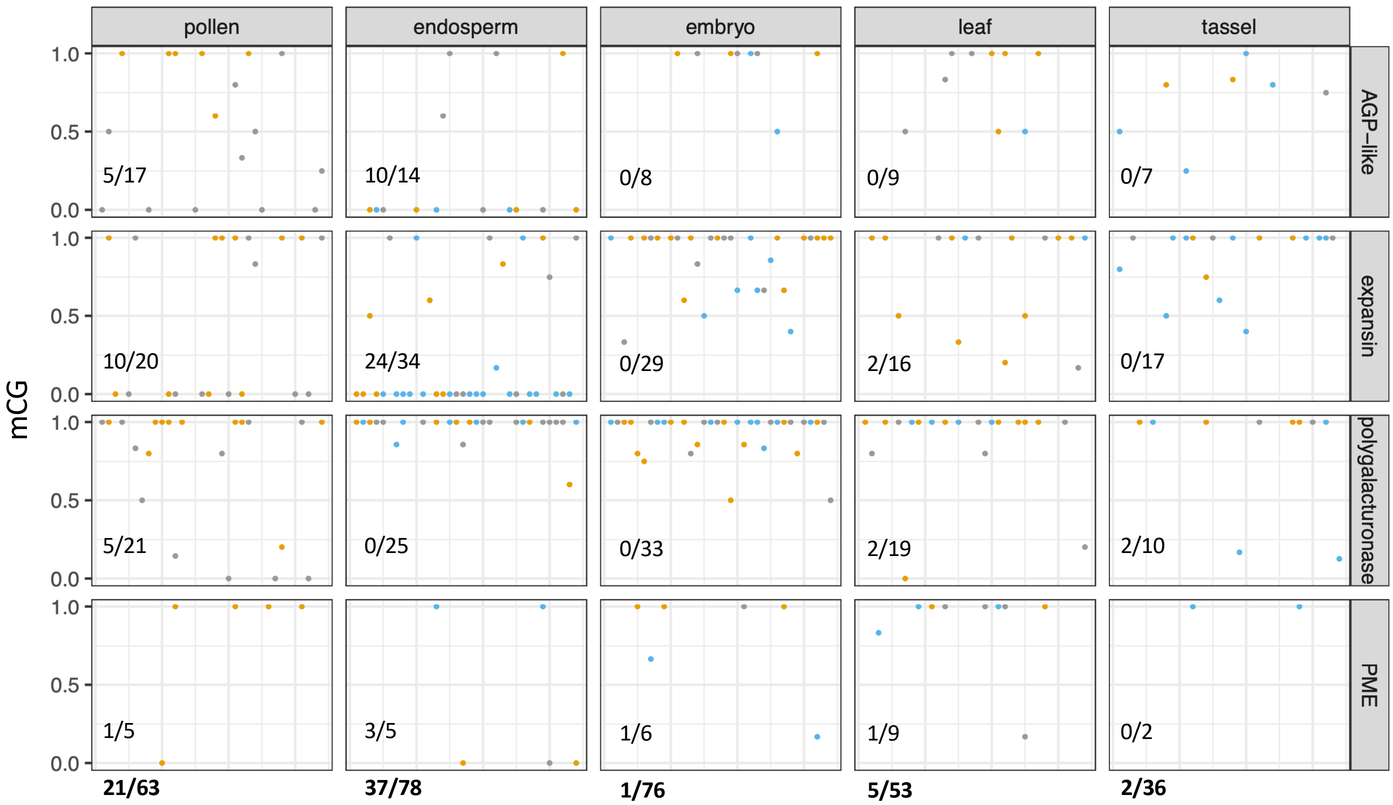

Supplementary Fig. 9: Demethylation of four representative promoters.
Single-read CG methylation calls from four MPG promoters. Each dot represents a read or read segment, biological replicates distinguished by color. All reads or segments of reads that overlapped 600 bp upstream of each gene in the W22 genome and which included at least four CGs were included in the analysis. The position on the X axis is randomly generated to prevent overlapping dots. The AGP-like gene corresponds to Zm00001eb316000, the expansin to Zm00001eb130160, the polygalacturonase to Zm00001eb132550, and the pectin methylesterase (PME) to Zm00001eb239290. Numbers in the bottom left of each panel indicate proportion of reads with less than or equal to CG methylation values of 0.2.

source tissue	library name	SRA run accession	150-nt read pairs	mCHH of lambda spike-in control
15-DAP endosperm	w1-EN	SRR18289859	45875343	0.20%
15-DAP endosperm	w2-EN	SRR18289858	51596713	0.19%
15-DAP endosperm	w3-EN	SRR18289847	47357875	0.22%
15-DAP embryo	w1-EM	SRR18289833	39632962	0.18%
15-DAP embryo	w2-EM	SRR18289832	41814310	0.18%
15-DAP embryo	w3-EM	SRR18289831	49590739	0.16%
second leaf	J664-leaf-rep1	SRR22950188	36592477	0.14%
second leaf	J664-leaf-rep2	SRR22950187	47888643	0.12%
second leaf	J664-leaf-rep3	SRR22950185	38451615	0.42%
premeiotic tassel	wt_J500s_EM-seq_rep1	SRR15725932	21951025	0.17%
premeiotic tassel	wt_J500s_EM-seq_rep2	SRR15725959	20640290	0.23%
premeiotic tassel	wt_J500s_EM-seq_rep3	SRR15725958	28257660	0.37%
pollen	MP-1	SRR29434332	72776554	0.86%
pollen	MP-2	SRR29434331	74454973	0.83%

Supplementary Table 1: EM-seq accession numbers, read counts, and conversion rates.
Endosperm and paired embryos were collected when embryos were ~3.5 mm x 2 mm in size. Second leaves were collected before unfurling of 1st leaf. Premeiotic tassels were collected when they were between 0.8 and 2.3 cm in length. Pollen was collected at fully mature stage, after release from anthers.
