# Supplementary material for: Potent pollen gene regulation by DNA glycosylases in maize
Source: Nat Commun. 2024 Sep 27;15:8352. doi: 10.1038/s41467-024-52620-y (PMC11436724; doi:10.1038/s41467-024-52620-y)
Supplement: Supplementary file 3 — Description of Additional Supplementary Files [file 41467_2024_52620_MOESM3_ESM.pdf]

<u>Description of Additional Supplementary Files</u>

Supplementary Data 1: DESeq2 stats and annotations for candidate DNG target genes. Gene IDs for each gene are from the B73 v5 annotation. Missing genes had no reads in any of the transcriptomes. baseMean, log2FoldChange, lfcSE, stat, pvalue, and padj values are from standard DESeq2 output. baseMean TPMs are calculated from WT and single mutant pollen transcriptomes combined. log2FoldChange is the fold change between single mutant and wild-type transcriptomes compared to double mutant transcriptomes. DEG means differentially expressed gene in comparison between mdr1 dng102 double mutant pollen and combined WT and single mutant pollen. MPG means methylated pollen gene, as defined by TElike methylation and anther specific expression.

Supplementary Data 2: CEL-seq barcodes and SRA accession numbers for single-pollen RNA-seq. Adapter sequences including 6-nt barcodes are listed for each of the 48 single-pollen transcriptomes, along with genotypes (if clear), and SRA accession numbers.

Supplementary Data 3: Gene names for B73 v4/v5 gene annotation conversions. Since expression timecourse data was from the B73 v4 annotation, corresponding v5 names are indicated for easy conversion.
